# MNQ derivative D19 alleviates LPS-induced inflammation and oxidative stress in sheep follicular granulosa cells through the *GPX4*-mediated ferroptosis

**DOI:** 10.3389/fvets.2025.1621738

**Published:** 2025-10-01

**Authors:** Chunlu Chen, Jia Lu, Yuhan Qu, Jianhua Dong, Yihao Hong, Yongping Ren, Shouqing Jiang, Rafael Baptista, Dong Wang, Luis Aj Mur, Lihua Lyu

**Affiliations:** ^1^College of Animal Science, Shanxi Agricultural University, Jinzhong, China; ^2^College of Software, Shanxi Agricultural University, Jinzhong, China; ^3^Institute of Biological, Environmental and Rural Sciences, Penglais Campus, Aberystwyth University, Aberystwyth, Wales, United Kingdom; ^4^Institute of Animal Science, Chinese Academy of Agricultural Sciences, Beijing, China; ^5^Department of Biology and Health, Aberystwyth University, Aberystwyth, United Kingdom

**Keywords:** lipopolysaccharide, granulosa cells, inflammation, oxidative stress, ferroptosis

## Abstract

**Introduction:**

2-methoxy-1,4-naphthoquinone (MNQ), a compound derived from *Impatiens balsamina L*., is recognized for its anti-inflammatory and antioxidant properties. However, the effects of D19, a derivative of MNQ, remain unexplored. This study aimed to elucidate the protective effect of D19 against lipopolysaccharide (LPS)-induced follicular granulosa cells (GCs) dysfunction in sheep and its underlying molecular mechanisms.

**Methods:**

An *in vitro* model of GCs injury was established using LPS to induce inflammation and oxidative stress. The effects of D19 were evaluated by examining inflammatory response, oxidative stress, ferroptosis and steroidogenesis following treatment. Gene interference was applied to knock down *GPX4* expression to validate its role in the protective mechanism of D19.

**Results:**

D19 attenuated LPS-induced ferroptosis in GCs by restoring the expression of the key ferroptosis regulator *GPX4*. Subsequently, interfering with *GPX4* activated NF-κB and upregulated the expression of inflammatory factors (*TNF-α*, *IL-1β*, *IL-6*) while disrupting NRF2 and inhibiting the expression of antioxidant-related factors (*CAT*, *GSH-PX*, *SOD2*). D19 effectively protected GCs from *GPX4* deficiency-induced inflammation and oxidative damage. Furthermore, D19 mitigated ferroptosis caused by *GPX4* deficiency and maintained iron metabolic homeostasis by restoring the morphology of GCs, increasing mitochondrial membrane potential, decreasing the accumulation of Fe^2+^ and lipid peroxides, and promoting the expression of *GPX4* and FTH1. D19 also improved steroid hormone secretion abnormalities caused by *GPX4* deficiency.

**Discussion:**

These results demonstrate that D19 protects sheep follicular GCs from LPS-induced damage by modulating the *GPX4*-mediated ferroptosis signaling pathway, providing new potential drugs and therapeutic targets for addressing GCs dysfunction and follicular developmental abnormalities.

## 1 Introduction

The global farming industry has historically prioritized enhancing productivity. However, a significant contemporary challenge confronting this sector is the prevalence of bacterial contamination, particularly affecting the reproductive system. Around 90% of animals develop uterine bacterial infections after parturition, leading to fertility decline, ovarian dysfunction, slower follicle development, and impaired steroidogenesis (1). Lipopolysaccharide (LPS), a significant component of Gram-negative bacteria, is a source of pathogenicity due to its release in large quantities during bacterial death or lysis and has been shown to cause inflammation, oxidative stress, and disruption of testosterone secretion in testicular macrophages of sheep (2). Pigs, mice, and humans exposed to bacterial contamination have shown higher concentrations of LPS in serum and follicular fluid (3). The causative agent of polycystic ovarian syndrome has also been found to be closely related to abnormal LPS levels (4).

As an essential component of the follicle, follicular granulosa cells (GCs) are responsible for providing nutrients to the oocyte and regulating steroid hormone synthesis, and their abnormal function is a major factor leading to follicular atresia. Extant data substantiate that LPS instigates aberrant expression of inflammatory factors, mitochondrial dysfunction, oxidative damage, and steroid-related hormone secretion disorders in follicular GCs, profoundly impacting the normal development of follicles (5). Consequently, based on the above research background, it is imperative to investigate the precise mechanism of LPS to develop effective therapeutic strategies for treating follicular atresia.

Ferroptosis is a unique mode of cell death, the initiation mechanism of which involves intracellular iron overload, ROS production, lipid peroxidation processes, mitochondrial membrane densification accompanied by volume reduction, rupture of the outer membrane, and reduction or disappearance of mitochondrial cristae, accompanied by a significant decrease in the content of reduced glutathione (GSH) and loss of the activity of glutathione peroxidase 4 (*GPX4*), which is significantly different from the traditional modes of cell death such as autophagy, apoptosis, and necroptosis. The GPX family is widely present in mammals, but its member, *GPX4,* is an indispensable antioxidant enzyme due to its unique amino acid sequence and spatial structure (6) and has been demonstrated to be an important target for the treatment of ferroptosis (7). *GPX4* deficiency compromises the antioxidant system in GCs, leading to intracellular oxidative stress, ferroptosis induction, and disruption of steroid hormone synthesis and secretion, ultimately resulting in abnormal ovulation (8, 9). The proteomic analysis revealed that LPS induced ferroptosis through the NRF2/GPX4 axis, accompanied by oxidative stress and inflammatory responses (10). Ferroptosis-mediated oxidative stress-inflammatory response, as evidenced by the down-regulation of NRF2/GPX4 and the up-regulation of NF-KB, has been identified in young rats presenting with premature ovarian failure (11). LPS inhibited the expression of *GPX4*, which activated NF-kB signaling and promoted the release of inflammation-associated factors *IL-1β*, *IL*-*6*, and *TNF-α*, as well as a large accumulation of intracellular ROS levels, whereas the intracellular GSH content was noticeably downregulated (12). In light of these findings, we hypothesize that LPS-induced inflammation and oxidative stress may occur through the *GPX4*-mediated ferroptosis pathway, severely limiting the reproductive efficiency of the animals. Therefore, there is an urgent need to find novel therapeutic interventions to address this issue.

Since the long-term use of conventional antibiotics can lead to problems such as drug resistance and drug residues within livestock products, more and more scholars are focusing on highly effective and low-toxicity natural herbal treatments. *Impatiens balsamina L.* contains a key active component, 2-methoxy-1,4-naphthoquinone (MNQ), which has been scientifically proven to possess a variety of bioactive properties, including antipruritic, anti-inflammatory, antimicrobial, anticancer, and antiallergenic effects (13). Available studies have shown that MNQ possesses potent antibacterial properties, effective against both Gram-positive (*Staphylococcus aureus*) and Gram-negative (*Escherichia coli*) bacteria, as well as *Helicobacter pylori* and several fungal species, such as *Penicillium* and *Fusarium* (14, 15). Treatment with MNQ promoted cell cycle progression from S phase to G2/M phase in olfactory ensheathing cells (OECs), stimulated mitotic division, enhanced proliferative capacity, and activated the NRF2-mediated antioxidant defense system (16). Previous studies have shown that MNQ mediates the TNF signaling pathway to alleviate inflammation and functional impairment in bovine follicular GCs and regulates steroid hormone synthesis (17).

**Figure 1:**
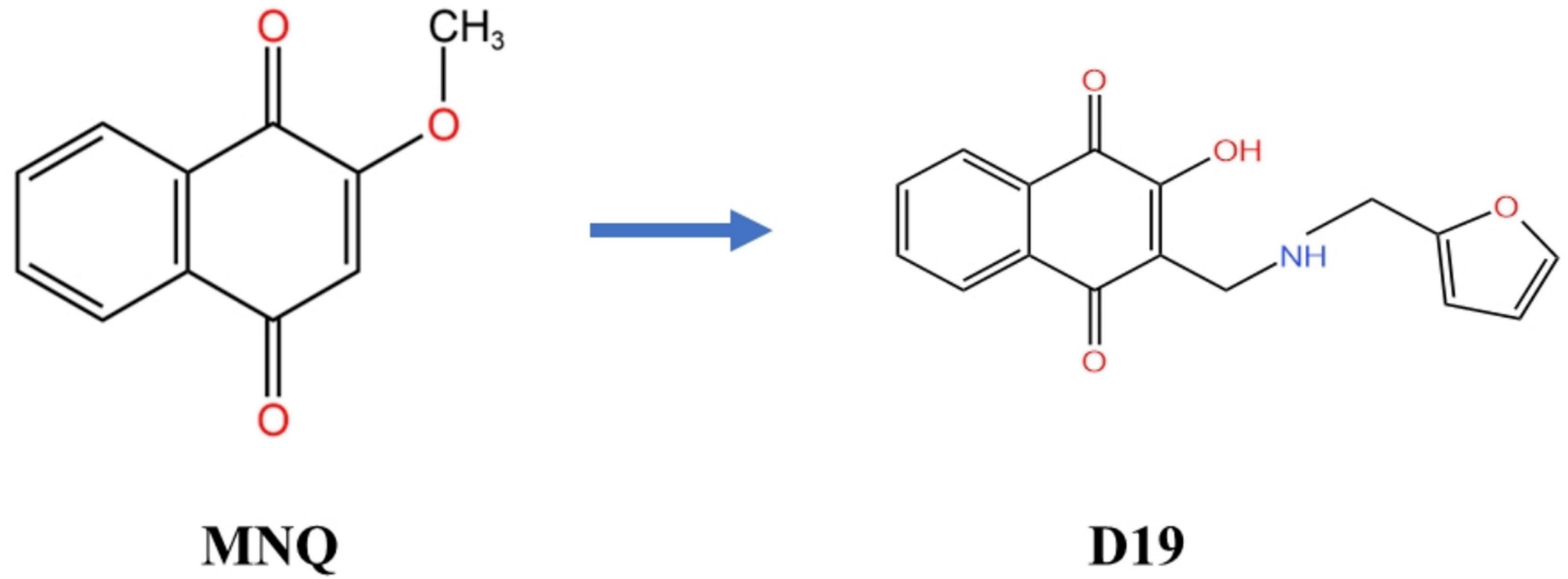
Molecular structures of MNQ and its derivative D19.

Based on the chemical structure of MNQ, we synthesized its derivative, D19, a compound whose functions have never been reported (Figure 1). Our team’s proteomic sequencing data suggests that the *GPX4*-mediated ferroptosis signaling pathway plays a key role in MNQ alleviation of LPS-induced functional impairment of GCs. Therefore, the present study aimed to explore whether *GPX4*-regulated ferroptosis is the underlying mechanism by which D19 exerts its protective effects against LPS-induced inflammation, oxidative stress, and steroid hormone synthesis disorders.

## 2 Materials and methods

### 2.1 Ovary collection and GCs cultivation

In this experiment, all sheep were grown to sexual maturity on the farm and transported to the local slaughterhouse for slaughter (Jinzhong, Shanxi, China). Meanwhile, we collected the ovaries on-site and preserved them in DPBS, sterilized at 4 °C with 100 IU/mL of penicillin and 100 mg/mL of streptomycin (Solarbio, Beijing, China), and brought them back to the laboratory. The ovaries were sterilized with 75% alcohol, and follicles with a diameter of 3–5 mm were selected to remove the GCs for *in vitro* culture. Specific culturing methods were carried out as previously described (18). MNQ was isolated from the stems and leaves of *Impatiens balsamina L*., and its derivative D19 was synthesized based on the molecular structure of MNQ following previously reported methods (19). The structure of D19 was characterized by ^1^H nuclear magnetic resonance (^1^H NMR, 400 MHz, DMSO-d6), with characteristic peaks observed at *δ* 8.53 (s, 1H), 7.97–7.89 (m, 1H), 7.82 (d, J = 7.6 Hz, 1H), 7.73–7.68 (m, 1H), 7.57 (t, J = 7.5 Hz, 1H), 6.71 (dd, J₁ = 14.7 Hz, J₂ = 3.3 Hz, 1H), 6.58–6.44 (m, 2H), 4.28 (s, 1H), 4.20–4.00 (m, 3H), and 3.95 (s, 1H).

### 2.2 Test groups for GCs

GCs were evenly suspended in a complete medium [89% DMEM-F12 (BOSTER, Wuhan, China), 10% fetal bovine serum (Cellmax, Beijing, China), and 1% penicillin–streptomycin (BOSTER, Wuhan, China)], inoculated in cell culture plates of different sizes and cultured in a 37 °C and 5% CO_2_ humidified incubator. The inoculum densities of GCs in 96-well and 6-well plates were 1 × 10^4^ cells/well and 1 × 10^6^ cells/well, respectively (20). GCs were treated with D19 (prepared as a 10 mM stock solution in DMSO; final DMSO concentration <0.1%) at concentrations of 0, 0.01, 0.1, 1, 10, 100, and 200 μM. Control groups (CK) received equivalent DMSO concentrations without D19. All groups were incubated for 24 h and 48 h, respectively (17, 21). GCs were incubated with different concentrations of LPS (0, 10, 25, 50, 100, 200, 400 μg/mL) (Solarbio, Beijing, China) for 12 h and 24 h (22). Control siRNA (si-NC: sense, 5′-UUCUCCGAACGUGUCACGUTT-3′; antisense, 5′-ACGUGACACGUUCGGAGAATT-3′) and GPX4-targeting siRNA (si-GPX4: sense, 5′-AAGAGUUCGCUGCUGGCUA-3′; antisense, 5′-UAGCCAGCAGCGAACUCUU-3′) were synthesized by Shanghai Sangon Biotech. GCs were transfected with 20 μM of either si-NC or si-GPX4 using an RNA transfection reagent (Sangon, Shanghai, China). Transfection was performed for 12, 24, 36, or 48 h. The experimental groups included: (1) CK, (2) LPS (cells treated with LPS for 12 h), (3) LPS + D19 (cells treated with LPS for 12 h, followed by D19 treatment for an additional 24 h), (4) si-NC (cells transfected with control siRNA for 36 h), (5) si-GPX4 (cells transfected with GPX4-targeting siRNA for 36 h), and (6) si-GPX4 + D19 (0.1 μM) (cells transfected with GPX4-targeting siRNA for 12 h, followed by D19 treatment for an additional 24 h).

### 2.3 Immunofluorescence

The immunofluorescence experiments were performed as previously described (23). Anti-FSHR (Bioworld, Minnesota, United States) and anti-GPX4 (Sangon Biotech, Shanghai, China) were used as primary antibodies, and goat anti-rabbit IgG (BOSTER, Wuhan, China) was used as the secondary antibody. The negative control group was incubated with PBS instead of the primary antibody. Specifically, GCs were inoculated into six-well plates, and the medium was removed and replaced with 4% paraformaldehyde fixation when the cells had fused to 60–70%, and GCs were permeabilized with 1% TritonX-100 (Solarbio, Beijing, China) after washing with PBS, followed by sealing with 1% BSA (Solarbio, Beijing, China). GCs were incubated with anti-FSHR and anti-GPX4 antibodies overnight at 4 °C. Hoechst 33342 was used to stain the nuclei of the cells, and the antigen–antibody conjugate reaction was performed with anti-rabbit IgG, and then placed under a fluorescence microscope to observe the specific protein expression.

### 2.4 MTT analysis

The toxic effects of different concentrations of D19 and LPS, as well as si-GPX4 on the cells at different time intervals, were performed in strict accordance with the instructions of the MTT kit (Solarbio, Beijing, China). GCs viability of each experimental group was calculated using the OD detected at 490 nm.

### 2.5 SA-β-galactosidase staining analysis

The senescence of GCs in each treatment group was detected according to the instructions of the SA-β-galactosidase (SA-β-Gal) staining kit (Beyotime, Shanghai, China). Specifically, GCs were removed from the culture and added to β-galactosidase staining fixative, fixed at room temperature for 15 min, washed with PBS, and then the prepared working solution (1% β-galactosidase staining solution A, 1% β-galactosidase staining solution B, 93% β-galactosidase staining solution C, and 5% X-Gal solution) was incubated overnight at 37 °C. The senescent cells were visualized under a light microscope to produce the Dark blue color results.

### 2.6 Glucose and LDH assays

Glucose content and LDH activity in treated GCs were assayed in strict accordance with the Glucose kit (Njjcbio, Nanjing, China) and Lactate dehydrogenase assay kit (Njjcbio, Nanjing, China).

### 2.7 Transmission electron microscopy

The experimental procedure was performed as previously described (24). Briefly, the treated GCs were pre-fixed with 3% glutaraldehyde for about 24 h and 1% osmium tetroxide for another 2 h; dehydrated and osmotically embedded according to the concentration gradient, and then stained after ultrathin sectioning using an ultrathin sectioning machine, and finally the images were captured and analyzed in the electron microscope.

### 2.8 Mitochondrial membrane potential assay

Cultured GCs (treated) complete medium was replaced with 2 μM JC-1 (MCE, Shanghai, China) and continued to be cultured in the medium for 15–20 min, washed with PBS, and then the staining results were observed by fluorescence microscopy (green fluorescence: Ex/Em = 510/527 nm; red fluorescence: Ex/Em = 585/590 nm).

### 2.9 Measurement of androstenedione, estradiol, and progesterone, TNF-α, and IL-1β using ELISA

The levels of steroid hormones (A_4_, E_2_, and P_4_) and inflammatory factors (TNF-β, IL-1β) in the treated GCs were measured according to the instructions of the ELISA kit (MEIMIAN, Jiangsu, China). Specific experimental steps were performed as previously described (25). Briefly, the diluted GCs supernatant was labeled with horseradish peroxidase (HRP), incubated for 1 h at a constant temperature of 37 °C, washed five times with washing solution, and then reacted with substrates A and B simultaneously for 15 min. The reaction was terminated by adding a termination solution. Then, the OD value at 450 nm was measured in each well, and the final concentration was calculated according to the standard curve. It is worth noting that the procedure was the same for all five assays, all concentrations were normalized to 2.5 × 10^6^ cells, and all experiments were repeated at least three times.

### 2.10 Antioxidant assays

The antioxidant capacity of GCs in different treatment groups was detected by using Nanjing Jianjian Bioengineering Institute kits (including SOD, GSH, GSH-PX, MDA, total antioxidant capacity, CAT, and ROS) and Lipid Peroxidation Assay Kit (BODIPY 581/591 C11) (Beyotime, Shanghai, China) and operated according to the specifications. All experiments were repeated at least three times.

### 2.11 Ferrous ion (Fe^2+^) detection

A ferrous ion content assay kit (Solarbio, Beijing, China) was used to measure Fe^2+^ levels in GCs after adding different treatments. The OD value at 593 nm was substituted into the standard curve to calculate the final results, and each set of experiments was repeated at least three times.

### 2.12 qRT-PCR analysis

Total RNA was extracted from GCs using the Mei5bio RNA extraction kit (Mei5bio, Beijing, China), and cDNA synthesis was performed according to the protocol provided with the PrimeScript RT Reagent Kit (Takara, Tokyo, Japan). Quantitative real-time PCR (qRT-PCR) was conducted on a Bio-Rad CFX instrument using the TransGen PerfectStart^®^ Green qPCR SuperMix kit (TransGen, Beijing, China), following the manufacturer’s instructions. Primer sequences were designed and synthesized by Shanghai Sangon Biotech (see Table 1 for details). The qRT-PCR protocol included an initial denaturation step at 95 °C for 60 s, followed by 40 cycles of amplification (95 °C for 30 s, 95 °C for 5 s, and annealing at the primer-specific Tm for 30 s) (25). *β-actin* was used as an internal control, and each experiment was performed in triplicate to ensure reproducibility. Relative gene expression levels were calculated using the 2^−ΔΔCt^ method.

**Table 1:** Primer sequences for real-time PCR.

Gene	Sequence (5′→3′)	Product size (bp)
*IL-6*	F: CAATCTGGGTTCAATCAGGCGA R: TGCTCTGCAACTCCATGACAG	130
*IL-1β*	F: CGTCTTCCTGGGACGTTTTAG R: CTGCGTATGGCTTCTTTAGGG	85
*TNF-α*	F: GTAGCCCACGTTGTAGCCAA R: TGAGGTAAAGCCCGTCAGTG	136
*NF-KB*	F: CTCCTGGAGCCTCAAACCTG R: TCTACAGGGAAAACTGAATCTTTCT	121
*SOD2*	F: GTGGAGAACCCAAAGGGGAAT R: GCAGCAATCTGTAAGCGTCC	160
*GPX4*	F: TCGCTGCTGGCTATAACGTC R: CCATTTGATGGCGTTTCCCA	134
*CAT*	F: GCCTTCTGCCCTGGAACATA R: TAGAAATCCCGCACCTGAGTG	96
*NRF2*	F: AAGTCAGGGAGAAGCGAGTTC R: TGTCAATCAAATCCATGTCCTGC	199
*FTH1*	F: GCCATCAACCGCCAGATCAA R: GAAACTCGGCTCCCATGGACA	70
*STAR*	F: GCATCCTCAAAGACCAGGAG R: CTTGACACTGGGGTTCCACT	194
*3β-HSD*	F: GGAGACATTCTGGATGAGCAG R: TCTATGGTGCTGGTGTGGA	200
*CYP11A1*	F: GTTTCGCTTTGCCTTTGAGTC R: ACAGTTCTGGAGGGAGGTTGA	158
*CYP19A1*	F: GCACTCTGGAAAGCTGTTCG R: CACGTCCACATAGCCCAAGT	147
*HSD17B4*	F: ACGTGTCGAGATTCAAGGCA R: CCAGTTCCTTGGACCTTGGTT	127
*β-actin*	F: GCAAAGACCTCTACGCCAAC R: GGGCAGTGATCTCTTTCTGC	90

### 2.13 Western blotting analysis

Total GC proteins were extracted with ready-to-use lysate (98% RIPA, 1% PMSF, 1% broad-spectrum phosphorylated protease inhibitor), centrifuged, and added to the protein sample buffer at a ratio of 4:1. After SDS-PAGE electrophoresis (80 V for 40 min, 120 V for 90 min), the proteins were transferred to NC membranes (120 V, ice bath for 90 min). The membrane was blocked with skimmed milk powder for 1 h, the primary antibody was incubated overnight at 4 °C, and the secondary antibody (LI-COR, Lincoln, NE, United States) was incubated at room temperature and protected from light for 1 h. The above reagents were purchased from BioTech. Protein bands were imaged using the Odyssey Infrared Imaging System and ImageJ to analyze relative changes. *β*-actin was used as an internal reference protein. Antibody information is shown in Table 2.

**Table 2:** Antibodies information.

Antibodies	Cat no.	Company	Source	Dilution ratio
IL-1β	D220820	Sangon	Rabbit	1:500
TNF-α	CPA9458	Cohesion biosciences	Rabbit	1:500
NF-KB	380172	Zenbio	Rabbit	1:500
pNF-KB	310013	Zenbio	Rabbit	1:500
GPX4	D290599	Sangon	Rabbit	1:1000
NRF2	380773	Zenbio	Rabbit	1:500
SOD2	CY5977	Abways	Rabbit	1:500
GSH-Px	CY8714	Abways	Rabbit	1:500
CAT	D122036	Sangon	Rabbit	1:500
FTH1	CY5648	Abways	Rabbit	1:1000
HSD17B4	D122505	Sangon	Rabbit	1:500
3β-HSD	CY8791	Abways	Rabbit	1:1000
CYP11A1	bs-10099R	Bioss	Rabbit	1:500
CYP19A1	bs-0114R	Bioss	Rabbit	1:500
STAR	A16432	ABclonal	Rabbit	1:500
β-actin	AC038	ABclonal	Rabbit	1:10000

### 2.14 Statistical analysis

Data were analyzed statistically using SPSS 22.0, subject to normality and chi-square: one-way ANOVA with Tukey’s-b or Dunnett’s *post-hoc* multiple comparisons between groups; *t*-tests for independent samples were used between groups. GraphPad Prism 8.0 was used for graphical presentation. Data are presented as mean ± SEM. *p* < 0.05 was the threshold for statistical significance, and all experiments were repeated at least three times.

## 3 Result

### 3.1 Ameliorative effect of D19 treatment on LPS-induced reduction in viability of GCs

**Figure 2:**
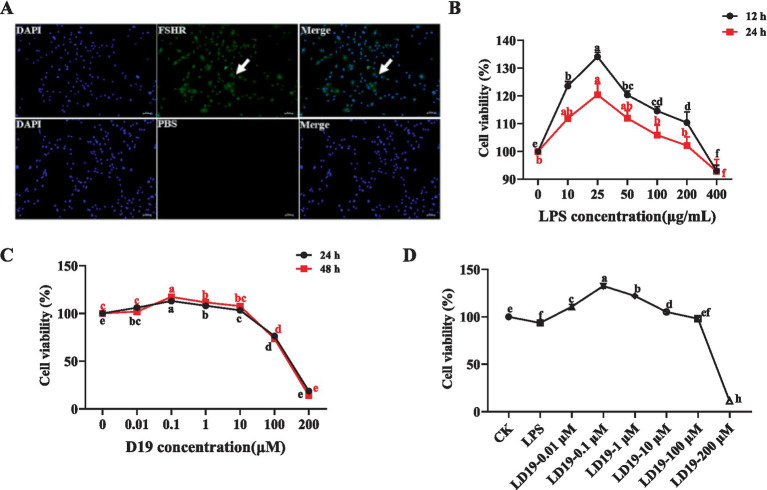
D19 treatment effectively mitigates the effects of LPS damage on the viability of GCs. **(A)** Cellular immunofluorescence showing FSHR-specific expression in GCs. **(B)** MTT assays were performed to evaluate cell viability following 12 and 24 h treatment with LPS (0, 10, 25, 50, 100, 200, and 400 μg/mL). **(C)** Cell viability was assessed by MTT assay after 24 and 48 h of D19 treatment (0, 0.01, 0.1, 1, 10, 100, 200 μM). **(D)** Mitigating the impact of different concentrations of D19 on LPS-induced decrease in cell viability of GCs. Data from at least three independent experiments are presented as mean ± SEM. Significant differences (*p* < 0.05) are denoted by different letters.

Immunofluorescence staining showed that the FSHR protein displayed particular expression in sheep follicle GCs, demonstrating that the GCs used in this study possess high purity and activity and are appropriate for subsequent experiments (Figure 2A).

Figure 2B demonstrates a concentration-dependent effect of LPS on GC viability, with the optimal survival rate observed at 25 μg/mL (*p* < 0.05). Then, the survival rate of the cells decreased with the increase in the concentration. When the concentration of LPS reached 400 μg/mL, the GCs were stressed, and some cells died. The results of GCs treated with different concentrations of D19 showed that low concentrations of D19 (0–0.1 μM) accelerated the activity of GCs (Figure 2C). The survival rate of GCs was significantly increased (*p* < 0.05) after treatment with 0.1 μM D19 for 24 h and 48 h. On the contrary, high concentrations (1–200 μM) of D19 caused dose-dependent cytotoxicity. Approximately 80% of GCs at 200 μM showed morphological abnormalities and were detached (*p* < 0.05). Since 400 μg/mL LPS would damage the activity of GCs, the present study was carried out to investigate the optimal therapeutic concentration and safety range of D19 at different concentrations. Our findings indicate that 0.1 μM D19 is the optimal therapeutic concentration, effectively mitigating LPS-induced damage in GCs (*p* < 0.05). However, D19 exhibited a narrow therapeutic window, with concentrations exceeding 100 μM failing to provide any protective benefit and instead demonstrating significant toxicity (Figure 2D).

### 3.2 D19 alleviates LPS-mediated inflammatory response

**Figure 3:**
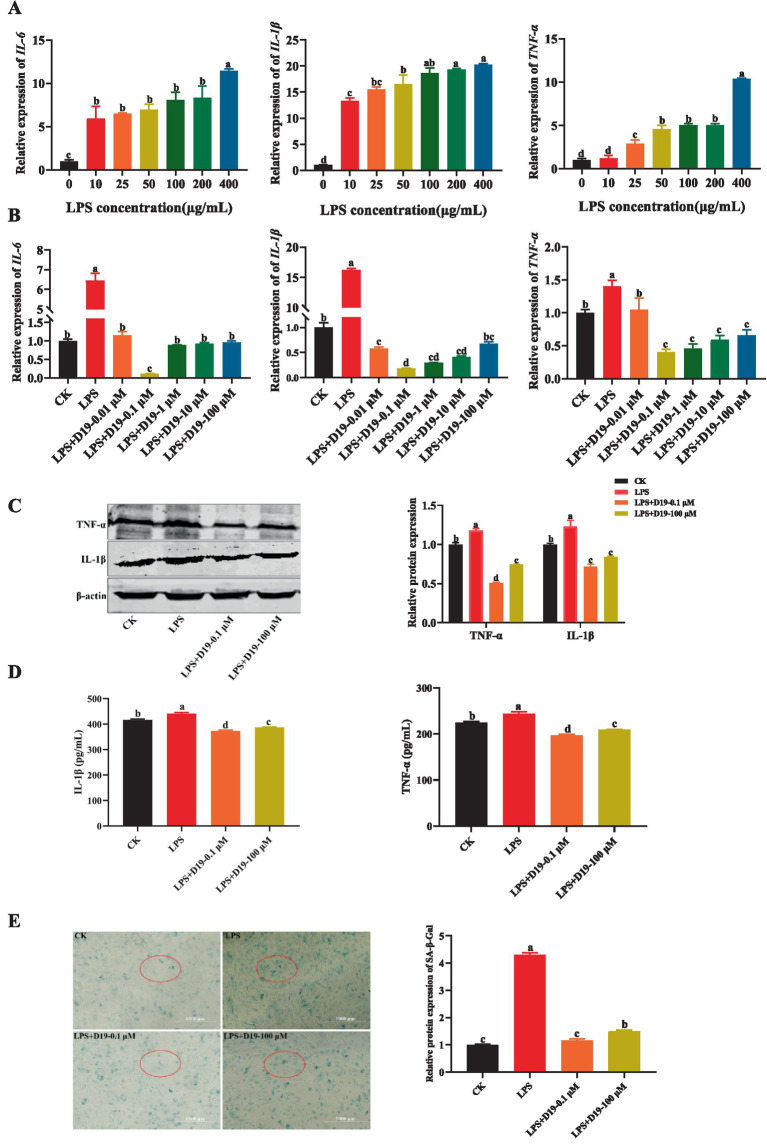
D19 alleviates LPS-mediated GCs inflammatory response. **(A)** Relative expression of *IL-6*, *IL-1β*, and *TNF-α* in GCs under different LPS concentrations. **(B)** Effects of varying concentrations of D19 on *IL-6*, *IL-1β*, and *TNF-α* levels induced by LPS (400 μg/mL). **(C)** Western blotting analysis of IL-1β and TNF-α protein expression in GCs. **(D)** ELISA quantification of IL-1β and TNF-α levels in different GCs treatment groups. **(E)** Senescence levels of GCs were assessed using the SA-β-Galactosidase Staining kit. Data originating from at least three independent experiments are presented as mean ± SEM. Significant differences (*p* < 0.05) are denoted by different letters.

To explore the mitigating effect of D19 on LPS-induced inflammation in GCs, the mRNA expression levels of *IL-6*, *IL-1β*, and *TNF-α* in GCs treated with different concentrations of LPS were first detected. The results showed that LPS dose-dependently increased the mRNA expression of the above inflammatory factors compared with CK (*p* < 0.05), with the maximum observed at a concentration of 400 μg/mL (Figure 3A). In subsequent experiments, 400 μg/mL LPS was used to induce inflammation in GCs. Further studies revealed that D19 was able to inhibit the LPS-induced inflammatory response of GCs. Compared with the LPS group, 0.1 μM D19 significantly reduced the mRNA levels of *IL-6*, *IL-1β*, and *TNF-α* (*p* < 0.05), and the therapeutic effect at this concentration was better than that of other concentrations (Figures 2D, 3B). In addition, 100 μM of D19 was the maximum safe concentration for treating inflammation in combination with cell activity assay (Figures 2D, 3B). D19 was found to significantly inhibit the LPS-induced elevation of IL-1β and TNF-α levels in GCs by ELISA kit assay (*p* < 0.05) (Figure 3D), and the results of protein levels were consistent with the above findings (Figures 3B,C). Notably, SA-β-Gal staining revealed that the dark blue products in GCs were significantly reduced in the LPS + D19-0.1 μM and LPS + D19-100 μM groups compared to the LPS group, indicating that D19 alleviated LPS-induced cellular senescence (Figure 3E). Moreover, the effect of D19 was more pronounced at 0.1 μM than at 100 μM (Figure 3E). The above studies confirmed that D19 reversed LPS-induced inflammatory injury in sheep follicular GCs.

### 3.3 D19 protects GCs from LPS-induced oxidative damage

**Figure 4:**
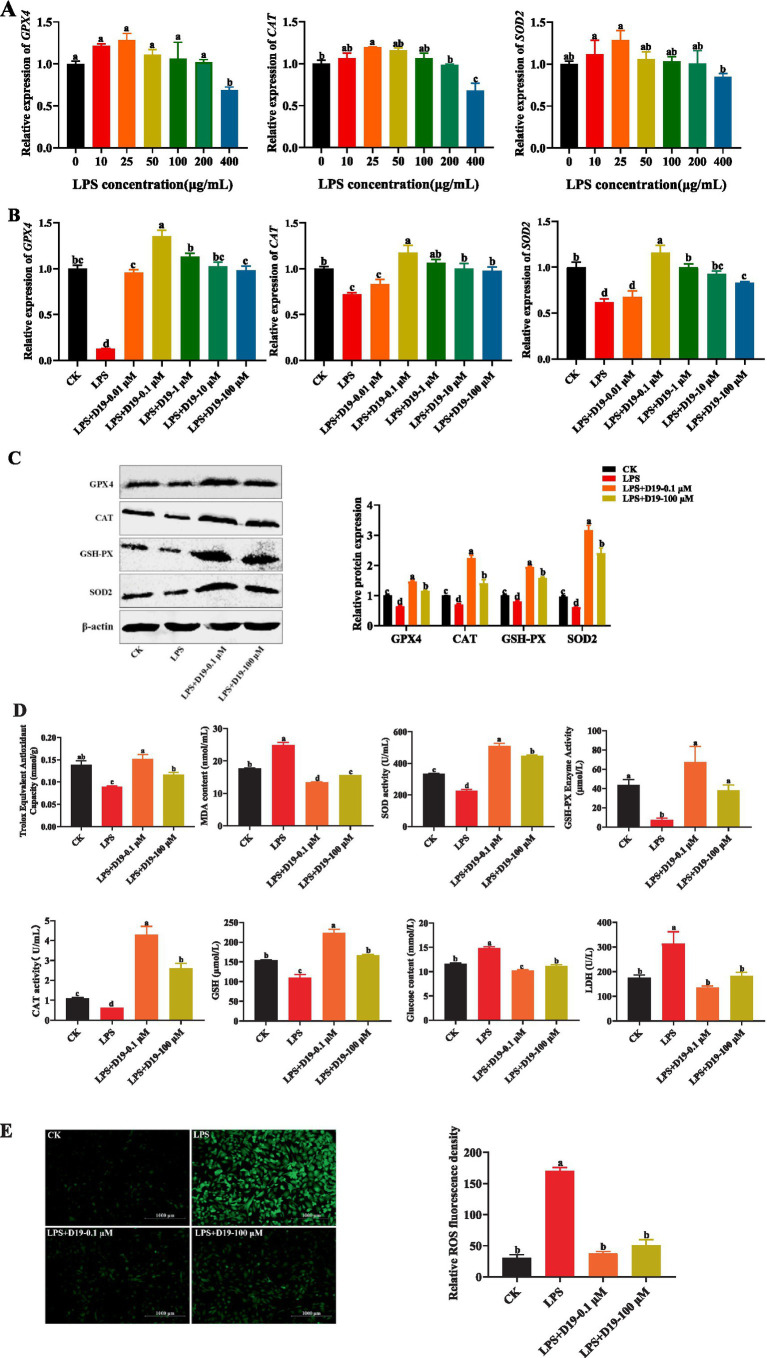
D19 protecting GCs from LPS-induced oxidative damage. **(A)** qRT-PCR analysis of relative *GPX4*, *CAT*, and *SOD2* mRNA expression in GCs exposed to different LPS concentrations. **(B,C)** The effects of varying concentrations of D19 on the mRNA and protein levels of *GPX4*, *CAT*, and *SOD2* induced by LPS (400 μg/mL) were analyzed using qRT-PCR and Western blotting. **(D,E)** Total antioxidant capacity, SOD activity, MDA levels, GSH-Px activity, CAT activity, GSH levels, glucose concentration, LDH levels, and ROS levels in GCs under different treatments, as measured using antioxidant assay kits. Data from at least three independent experiments are presented as mean ± SEM. Significant differences (*p* < 0.05) are denoted by different letters.

As shown in Figure 4A, 400 μg/mL LPS significantly inhibited the expression of antioxidant-related genes *GPX4*, *CAT*, and *SOD2* in sheep follicular GCs (*p* < 0.05). D19-0.1 μM effectively upregulated the expression levels of the genes mentioned above, with its effective concentration extending up to 100 μM (*p* < 0.05) (Figure 4B). Western blotting analysis (Figure 4C) confirmed that 0.1 μM D19 could effectively alleviate the LPS-induced reduction in the protein expression of GPX4, CAT, GSH-PX, and SOD2 (*p* < 0.05). 100 μM D19 also had antioxidant effects, but the effect was weaker than that of 0.1 μM (*p* < 0.05). In addition, 400 μg/mL LPS effectively inhibited the total antioxidant capacity, SOD activity, GSH-PX activity, CAT activity, and GSH level of GCs, and significantly increased MDA content, glucose content, LDH level, and ROS production. D19 could significantly reverse the above LPS-induced weakening of antioxidant indexes in GCs (Figures 4D,E). These data demonstrate that D19 protects sheep follicular GCs from LPS-induced oxidative damage.

### 3.4 D19 inhibits LPS-induced ferroptosis in GCs

**Figure 5:**
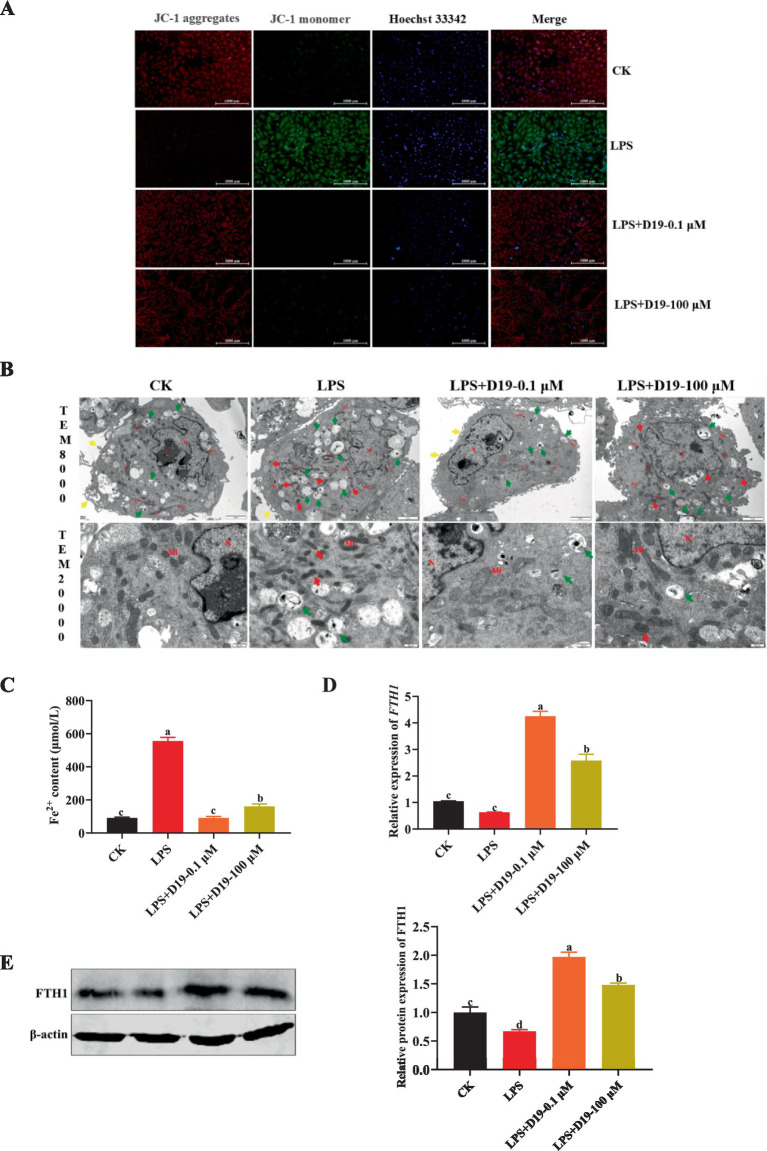
D19 inhibiting LPS-induced ferroptosis in GCs. **(A)** The effect of D19 on LPS-induced mitochondrial membrane potential changes was assessed using JC-1 staining. **(B)** TEM was used to examine the ultrastructural morphology of GCs in different treatment groups. Nucleus (N), nucleolus (No), mitochondria (Mi), rough endoplasmic reticulum (RER); microvilli (↑, yellow arrows), autolysosomes (↑, green arrows). Scale bars: TEM × 8,000, 2 μm; TEM × 20,000, 500 nm. **(C–E)** The effects of D19 (0.1 μM, 100 μM) on LPS-induced changes in ferrous ion levels and *FTH1* gene and protein expression were evaluated. Data from at least three independent experiments are presented as mean ± SEM. Significant differences (*p* < 0.05) are denoted by different letters.

Given that *GPX4*, a core molecule in ferroptosis regulation, has been significantly inhibited by LPS in this study, and combining the above findings, we speculate that the protective effect of D19 against LPS-induced functional impairment of GCs may be realized through the *GPX4*-mediated ferroptosis signaling pathway. As depicted in Figure 5A, LPS treatment significantly decreased the mitochondrial membrane potential, as evidenced by enhanced green fluorescence, whereas the mitochondrial membrane potential in the LPS + D19-0.1 μM and LPS + D19-100 μM groups was close to the normal level. TEM results (Figure 5B) revealed that GCs in the LPS-treated group displayed significant ultrastructural abnormalities compared with the CK group. These abnormalities included mitochondrial condensation, reduced volume, reduction and coarsening of cristae, widening of the inter-cristae lumen, and increased membrane and electron densities. Furthermore, autophagic lysosomes and a small number of vacuoles were observed. Compared with the LPS group, LPS + D19-0.1 μM significantly improved mitochondrial morphology, with most of the mitochondria restored to an elliptical shape, uniform matrix electron density, and well-defined and straight cristae. The morphology of LPS + D19-100 μM cells was slightly abnormal, and the mitochondria showed a slight condensation, accompanied by a mild expansion of the rough endoplasmic reticulum. Autophagic lysosomes were still visible in the cytoplasm. In addition, the large accumulation of Fe^2+^ in LPS-treated cells was significantly reversed by D19 treatment (Figure 5C), which also successfully alleviated the inhibitory effect of LPS on *FTH1* mRNA and protein expression levels (*p* < 0.05) (Figures 5D,E). Taken together, these results suggest that D19 can effectively alleviate the process of LPS-induced ferroptosis in sheep follicular GCs and mitigate cellular damage.

### 3.5 D19 restores LPS-suppressed steroid hormone production in GCs

**Figure 6:**
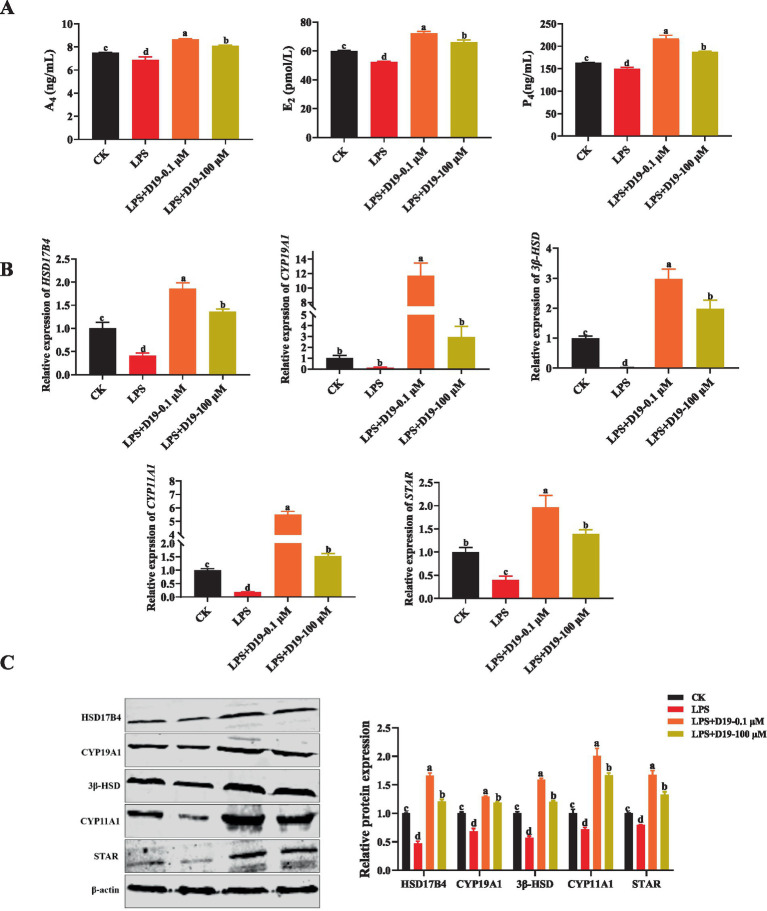
D19 restoring LPS-suppressed steroid hormone production in GCs. **(A)** ELISA measurement of A_4_, E_2_, and P_4_ levels in GCs. **(B,C)** qRT-PCR and Western blotting were employed to detect the relative expression levels of mRNA and protein, respectively, for genes involved in steroid hormone synthesis (*HSD17B4*, *CYP19A1*, *3β-HSD*, *CYP11A1*, and *STAR*). Data from at least three independent experiments are presented as mean ± SEM. Significant differences (*p* < 0.05) are denoted by different letters.

To assess the effect of D19 on LPS-induced steroid hormone synthesis in GCs, we examined the levels of A_4_, E_2_, and P_4_ in GCs. The ELISA results showed that LPS significantly inhibited the synthesis of these three hormones in GCs compared to CK (*p* < 0.05), whereas D19 treatment effectively reversed the inhibitory effect of LPS and significantly elevated the A_4_, E_2_, and P_4_ expression levels (*p* < 0.05) (Figure 6A). The expression of steroid synthesis-related genes (*HSD17B4*, *CYP19A1*, *3β-HSD*, *CYP11A1*, and *STAR*) and their proteins were also greatly inhibited by LPS, and the expression levels of these genes and proteins were significantly increased by D19 treatment (*p* < 0.05) (Figures 6B,C). Based on these findings, we conclude that D19 effectively mitigates LPS-induced impairment of steroid hormone synthesis in sheep follicular GCs.

### 3.6 D19 suppresses inflammation caused by *GPX4* deficiency in GCs

**Figure 7:**
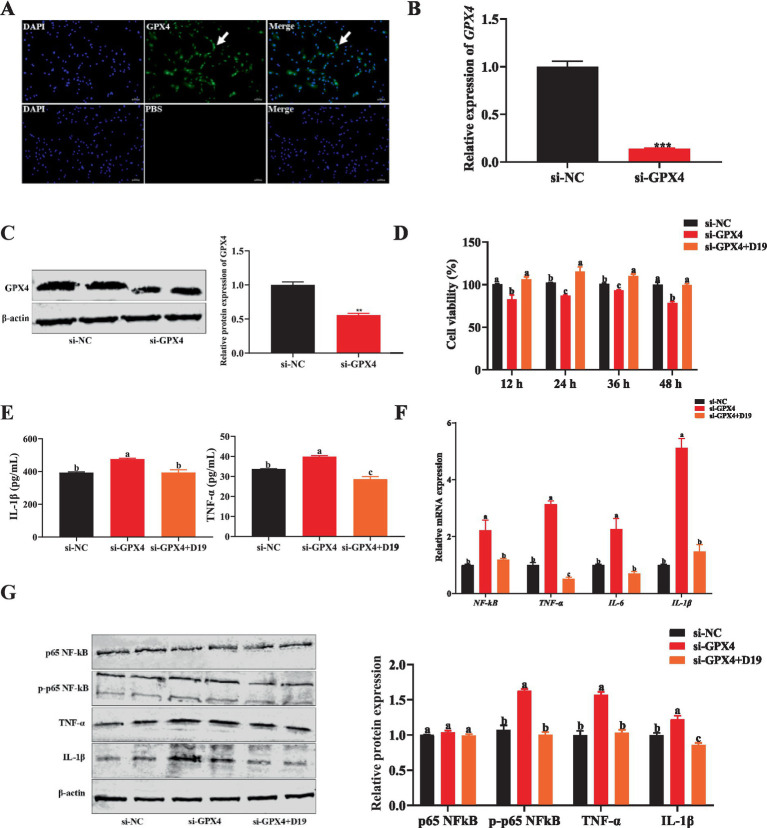
D19 suppressing inflammation caused by *GPX4* deficiency in GCs. **(A)** Immunofluorescence was utilized to detect the expression of GPX4 protein in GCs. **(B,C)** qRT-PCR and Western blotting were used to analyze the interference efficiency of si-GPX4. **(D)** D19–0.1 μM reversed the si-GPX4-induced decline in the cell viability of GCs. **(E)** ELISA measurement of IL-1β and TNF-α levels in treated GCs. **(F)** Relative mRNA expression of *NF-κB*, *TNF-α*, *IL-6*, and *IL-1β* in GCs, as determined by qRT-PCR. **(G)** Western blotting was conducted to investigate the effect of D19 on LPS-induced changes in the protein levels of p65 NF-κB, phosphorylated p65 NF-κB (p-p65 NF-κB), TNF-α, and IL-1β in GCs. Data are expressed as mean ± SEM (*n* ≥ 3). Different letters indicate significant differences at *p* < 0.05, while ** and *** denote *p* < 0.01 and *p* < 0.001, respectively.

GPX4 protein is specifically expressed in sheep follicular GCs (Figure 7A). si-GPX4 effectively inhibited *GPX4* mRNA and protein expression (*p* < 0.05) (Figures 7B,C) and significantly reduced cell viability at multiple time points (*p* < 0.05) (Figure 7D). However, 0.1 μM D19 effectively alleviated the inhibitory effect of si-GPX4 on GC activity (*p* < 0.05) (Figure 7D). To further study the effect of *GPX4* deletion on the inflammatory response of GCs and to clarify the role of D19, we obtained the following findings: ELISA results (Figure 7E) revealed that D19 effectively reversed the si-GPX4-mediated upregulation of IL-1β and TNF-α levels in GCs (*p* < 0.05). Mechanistically, we found that si-GPX4 activated the NF-κB signaling pathway, indicated by a significant increase in *NF-κB* mRNA expression and p65 phosphorylation, subsequently promoting the expression of *TNF-α*, *IL-6*, and *IL-1β* (*p* < 0.05) (Figures 7F,G). Notably, total p65 protein levels remained unchanged across all groups (*p* > 0.05) (Figure 7G). The protein levels of TNF-α and IL-1β were consistent with those of mRNA (*p* < 0.05) (Figures 7F,G). Interestingly, after D19 treatment, the expression levels of *NF-κB*, *TNF-α*, *IL-6*, and *IL-1β* were significantly lower than those in the si-GPX4 group (*p* < 0.05) and returned to near-normal cell levels. Meanwhile, Western blotting results showed that the trends of protein levels of p65 NF-κB, TNF-α, and IL-1β were consistent with the mRNA results (Figure 7G). Therefore, we conclude that D19 can effectively alleviate si-GPX4-induced decrease in cell viability and inflammatory injury.

### 3.7 Effect of D19 on oxidative damage in GCs induced by *GPX4* deficiency

**Figure 8:**
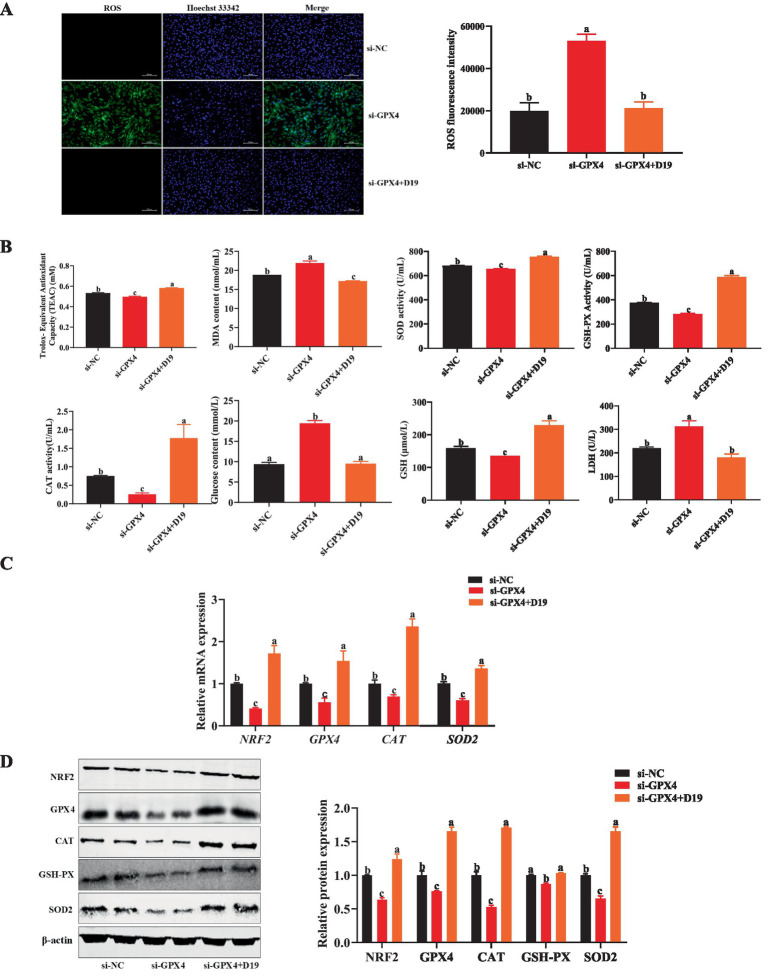
Effect of D19 on oxidative damage in GCs induced by *GPX4* deficiency. **(A,B)** D19 alleviated the effects of LPS on antioxidant-related indicators. **(C)** Relative mRNA expression of *NRF2*, *GPX4*, *CAT*, and *SOD2* in GCs from different treatment groups, as determined by qRT-PCR. **(D)** Relative protein expression of NRF2, GPX4, CAT, GSH-Px, and SOD2 in GCs from different treatment groups, as determined by Western blotting. Data are presented as mean ± SEM (*n* ≥ 3). Different letters denote significant differences (*p* < 0.05).

To investigate whether D19 protects against LPS-induced oxidative damage via *GPX4*, we suppressed *GPX4* expression in sheep follicular GCs and assessed oxidative stress and D19’s effects. Compared with GCs transfected with si-NC, antioxidant indices (total antioxidant capacity, SOD activity, CAT activity, GSH-PX activity, and GSH level) were significantly decreased (*p* < 0.05), while oxidative damage and metabolism-related indices (ROS level, MDA content, glucose level, and LDH activity) were significantly increased (*p* < 0.05) in GCs with *GPX4* knockdown (Figures 8A,B). Si-GPX4 significantly reduced mRNA and protein expression levels of antioxidant-related genes (*NRF2*, *CAT*, *SOD2*, and *GPX4*) compared to si-NC (*p* < 0.05), as shown in Figures 8C,D. After the D19 treatment, the above antioxidant indexes were significantly restored (Figure 8B), oxidative damage and metabolism-related indexes were reduced (Figures 8A,B), and the expression levels of antioxidant-related genes and proteins were effectively up-regulated (*p* < 0.05) (Figures 8C,D). These data demonstrate that D19 has therapeutic potential for treating si-GPX4-induced oxidative damage in GCs.

### 3.8 D19 mediates ferroptosis in GCs by regulating *GPX4*

**Figure 9:**
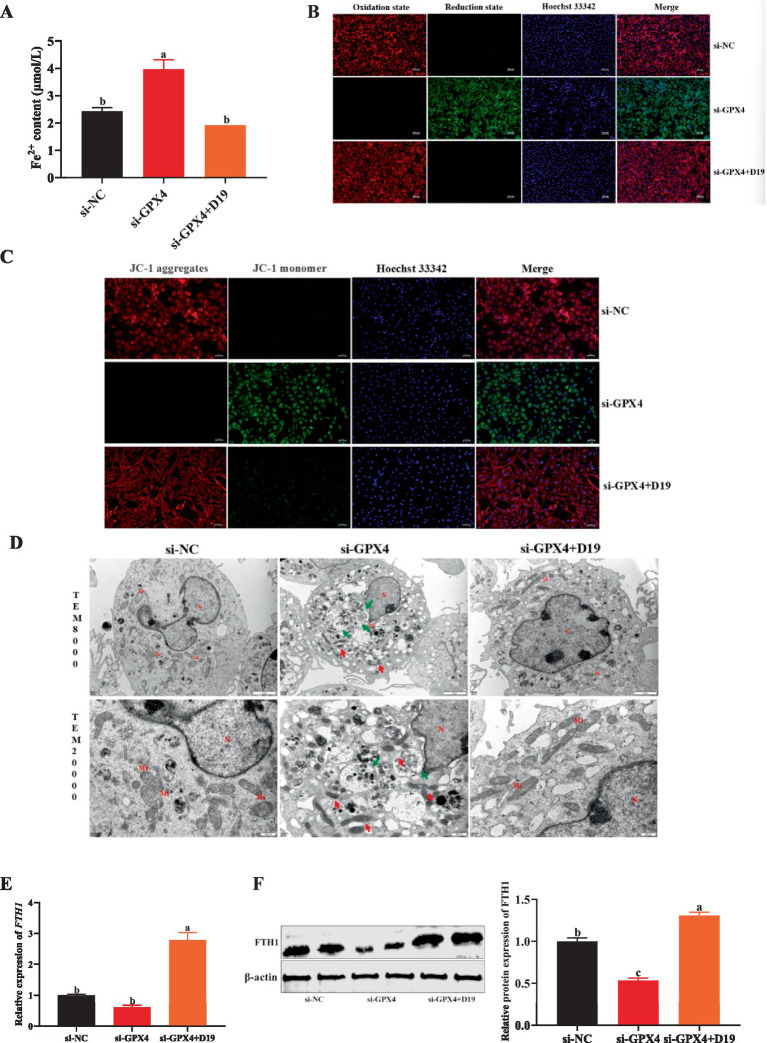
D19 mediates ferroptosis in GCs by regulating *GPX4*. **(A,B)** D19 protects GCs from LPS-induced abnormalities in Fe^2+^ and lipid peroxide levels. **(C,D)** Mitochondrial membrane potential and cellular ultrastructure of GCs in different treatment groups were assessed using JC-1 staining and TEM, respectively. Nucleus (N), mitochondria (Mi), autolysosomes (↑ green arrows). Scale bars: TEM × 8,000, 2 μm; TEM × 20,000, 500 nm. **(E,F)** Relative mRNA and protein expression levels of *FTH1* in GCs from different treatment groups. All experiments were performed in triplicate, and data are presented as mean ± SEM. Different letters indicate statistically significant differences (*p* < 0.05).

Evidence indicates that intracellular inflammatory responses and oxidative stress are strongly linked to ferroptosis caused by *GPX4* downregulation. Thus, we investigated whether D19 alleviates *GPX4* deficiency-induced inflammation and oxidative damage in GCs by modulating ferroptosis. Suppressing *GPX4* dramatically elevated Fe^2+^ concentration in GCs compared to the si-NC group (*p* < 0.05), as demonstrated in Figure 9A. However, D19 treatment in si-GPX4-transfected GCs normalized Fe^2+^ levels, indicating D19’s ability to rescue GCs from *GPX4* deficiency-induced iron overload (see Figure 9A). As shown in Figure 9B, si-GPX4-transfected GCs exhibited a significantly increased green fluorescence signal compared to the si-NC group, indicating elevated lipid peroxide levels. D19 treatment attenuated this increase, resulting in a red fluorescence signal distinct from the si-GPX4 group, suggesting that D19 effectively reduced lipid peroxide levels. TEM analysis showed that si-GPX4 GCs exhibited significant mitochondrial dysfunction, indicated by decreased membrane potential, cristae loss, increased membrane density, and elevated autophagosome numbers. D19 treatment mitigated these abnormalities, restoring mitochondrial morphology and membrane potential (Figures 9C,D). In addition, the gene and protein expression of *FTH1* in the GCs of the si-GPX4 group was significantly lower than that of the si-NC group (*p* < 0.05), whereas the expression level of *FTH1* in the si-GPX4 + D19 group was significantly higher than that of the si-GPX4 group (*p* < 0.05) (Figures 9E,F). Therefore, we conclude that D19 can effectively inhibit the ferroptosis process induced by *GPX4* deficiency and maintain the iron metabolism homeostasis in GCs.

### 3.9 D19 prevents *GPX4* deficiency-induced disruption of steroidogenesis in GCs

**Figure 10:**
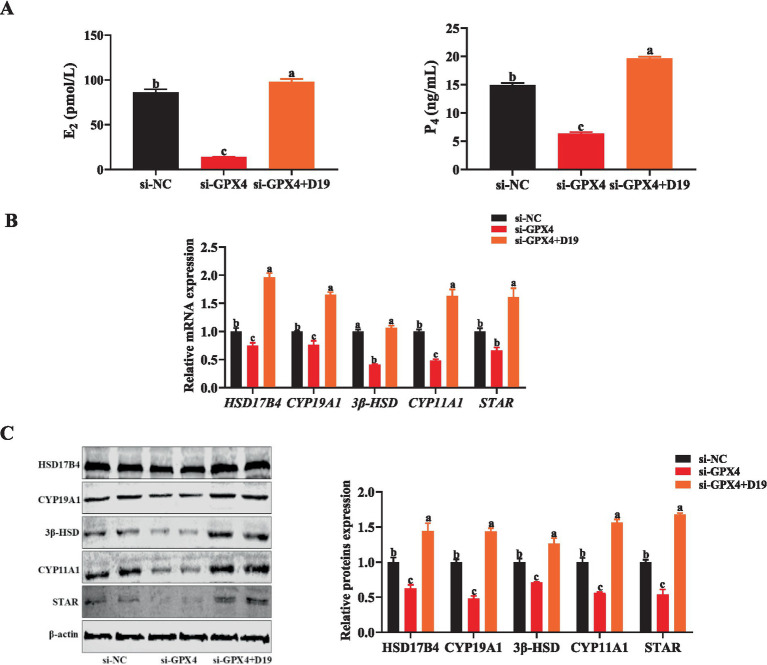
D19 preventing *GPX4* deficiency-induced disruption of steroidogenesis in GCs. **(A)** Levels of E_2_ and P_4_ in different treatment groups were measured using ELISA. **(B)** The relative mRNA and protein expression levels of steroid hormone synthesis-related genes (*HSD17B4*, *CYP19A1*, *3β-HSD*, *CYP11A1*, and *STAR*) were detected by qRT-PCR and Western blotting, respectively. All experiments were performed in triplicate, and data are presented as mean ± SEM. Different letters indicate statistically significant differences (*p* < 0.05).

The critical role of GCs in follicular development is realized through the synthesis of steroid hormones. However, when GCs were exposed to an environment in which *GPX4* was inhibited, the synthesis of E_2_ and P_4_ was significantly suppressed compared with the si-NC group (*p* < 0.05) (Figure 10A), while the mRNA and protein expression levels of steroid hormone synthesis-associated factors (*HSD17B4*, *CYP19A1*, *3β-HSD*, *CYP11A1*, and *STAR*) were also significantly downregulated (*p* < 0.05) (Figures 10B,C). Noticeably, the levels of E_2_ and P_4_, as well as the expression of steroid hormone synthesis-related genes and proteins, were significantly up-regulated in the si-GPX4 + D19 group compared with the si-GPX4 group (*p* < 0.05) (Figures 10A–C). These results suggest that D19 can effectively alleviate the inhibition of steroid hormone synthesis function in GCs by *GPX4* deletion and thus repair the functional damage of GCs.

## 4 Discussion

Dysfunction of follicular GCs in sheep is one of the key factors affecting normal follicular development and ovulation. LPS has been shown to induce inflammatory responses, oxidative stress, and steroid hormone synthesis disorders in GCs, leading to impaired GCs function. Increasing evidence demonstrates that the exposure of animals to LPS induces a range of reproductive disorders. Higher levels of LPS were detected in the blood, follicular fluid, and milk of cows with endometritis (26), accompanied by diminished ovarian function (27). Several studies have indicated that LPS not only inhibits GCs’ proliferation (28) but also triggers an inflammatory response *in vitro* in human follicular GCs, characterized by the upregulation of inflammatory cytokines, including *TNF-α*, *IL-1β*, and *IL-6* (29). LPS also significantly increased SA-β-gal activity, a cellular senescence marker, in macrophages (30). MNQ, belonging to the naphthoquinones class of compounds, is considered the most principal and representative active component of *Impatiens balsamina L.* Studies have confirmed that MNQ exhibits dose-dependent cytotoxicity, effectively killing various cancer cells at high concentrations while displaying anti-inflammatory and antioxidant activities at low concentrations (31). MNQ also promotes the proliferation of OECs at concentrations ranging from 0 to 1 μM, whereas a concentration of 10 μM is highly cytotoxic, significantly reducing cell viability (16). Our results demonstrate that low concentrations of D19 (0–100 μM) significantly ameliorated the LPS-induced decrease in GCs viability, whereas high concentrations of D19 exhibited toxicity similar to that of MNQ (32). Furthermore, MNQ has been reported to participate in host defense and exert immunomodulatory effects (33), as well as exhibit anti-neuroinflammatory activity by reducing nitric oxide (NO) production in LPS-stimulated BV-2 cells (34). Naphthoquinones extracted from *Sinningia canescens* effectively reduced the elevation of inflammatory factors TNF-α, IL-6, and IL-1β in mice blood induced by LPS (35). Consistent with these findings, our results demonstrate that the MNQ derivative D19 also exerts anti-inflammatory effects by downregulating the LPS-induced expression of the inflammation-related genes *TNF-α*, *IL-6*, and *IL-1β* in sheep follicular GCs.

LPS is recognized as a potent inducer of both inflammation and oxidative stress in cellular models *in vitro*. LPS led to a high accumulation of ROS in lung epithelial cells, thereby triggering oxidative stress (36). LPS up-regulated MDA levels and down-regulated antioxidant capacity in human follicular GCs, as well as inhibited the expression of *SOD*, *CAT*, and *GPx* genes (5). Interestingly, we observed a similar phenomenon in sheep follicular GCs: LPS significantly suppressed total antioxidant capacity, SOD activity, GSH-PX activity, and CAT activity, and reduced GSH levels in GCs, while increasing MDA and ROS levels. In addition, the transcriptional and translational levels of *CAT*, *GSH-PX*, and *SOD2* were also greatly inhibited by LPS, whereas the levels of LDH and glucose were significantly increased, a result that is consistent with the findings in BEAS-2B cells (37). The above results suggest that LPS can induce oxidative stress in sheep follicular GCs. Studies indicate that naphthoquinones (plumbagin, juglone, menadione, etc.) possess strong antioxidant capabilities by scavenging ROS and inducing the expression of antioxidant genes like *SOD* (38). Previous research has revealed potent antioxidant activity in certain extracts of *Impatiens balsamina* L. (39). However, studies on MNQ and its derivatives mainly focus on anticancer, antibacterial, and anti-inflammatory effects, while their antioxidant effects in animals are more limited (40). Interestingly, our experimental results revealed that D19 treatment significantly mitigated the LPS-induced impairment of antioxidant capacity in GCs, contributing to a more robust antioxidant defense and enriching the understanding of MNQ and its derivative D19’s antioxidant effects.

Steroid hormones are key regulators in maintaining follicular development and normal ovulation of oocytes. Within the steroid hormone synthesis pathway, P_4_ is converted to A_4_, which is further converted to E_2_, and these hormones collectively regulate the overall process (41). *CYP19A1* has been widely recognized as a key enzyme in estrogen synthesis (42). Previous studies have demonstrated that LPS-induced suppression of *CYP19A1* expression in bovine (43), mouse, and porcine follicular GCs leads to reduced E_2_ secretion and disruption of oocyte meiotic progression (44). *CYP11A1*, *3β-HSD*, and *STAR* are involved in the early stages of steroid hormone synthesis, where *CYP11A1* catalyzes the conversion of cholesterol to pregnenolone, and *STAR* is responsible for the transport of cholesterol to androgens. *STAR* is responsible for transporting cholesterol into mitochondria for *CYP11A1* utilization (22). Exposure of human follicular GCs to LPS also leads to a significant reduction in E_2_ and P_4_ levels, accompanied by a significant down-regulation of *CYP19A1*, *CYP11A1*, and *STAR* gene expression (45). *HSD17B4* is reported to function in testosterone synthesis by catalyzing the conversion of androstenedione into testosterone (46). Therefore, the reduction of these key enzymes inevitably leads to an overall decrease in steroid hormone synthesis. The results of the present study are consistent with previous studies, which found that exposure of sheep follicular GCs to LPS significantly reduced the gene and protein expression levels of *HSD17B4*, *CYP19A1*, *CYP11A1*, *3β-HSD*, and *STAR*, resulting in significant inhibition of E_2_, P_4_, and A_4_ synthesis. Our team’s previous research results showed that MNQ derivative D21 could alleviate the steroid hormone synthesis disorder caused by LPS (21). Encouragingly, the present study demonstrated that D19 exhibits a similar effect to D21 in mitigating LPS-induced steroid hormone synthesis disorder, further expanding the potential application of MNQ derivatives in enhancing follicular GCs function.

*GPX4*, a key negative regulator of the ferroptosis pathway, effectively inhibits ferroptosis through GSH-dependent scavenging of lipid peroxides and is a potential target for the treatment of ferroptosis-related diseases (11). Previous studies have shown that LPS inhibits *GPX4* expression in BEAS-2B cells, leading to intracellular Fe^2+^ accumulation and disruption of iron metabolic homeostasis (47). In addition, lower-than-normal levels of *GPX4* and *FTH1* in both human and mouse ovarian tissues from patients with ovarian cysts suggest ferroptosis (48). In the present study, we similarly observed that LPS significantly inhibited the expression of *GPX4* and *FTH1* in sheep follicular GCs, leading to Fe^2+^ accumulation, which was significantly reversed by D19 treatment. Following D19 treatment, the mRNA and protein expression levels of *GPX4* and *FTH1* in GCs were markedly increased compared to the LPS group, alleviating LPS-induced iron overload and further suppressing the ferroptosis process in GCs. Researchers have found that LPS induced mitochondrial morphological abnormalities and a decline in membrane potential in HT-22 cells (10). Interestingly, we also observed that D19 significantly protected sheep follicular GCs from LPS-induced mitochondrial damage and loss of membrane potential. Taken together with our team’s previous sequencing data, we speculate that the core mechanism by which D19 alleviates LPS-induced inflammation, oxidative stress, and steroid hormone synthesis disorders in sheep follicular GCs may be closely related to the *GPX4*-mediated ferroptosis signaling pathway.

It has been reported that *GPX4* is recognized as a key inhibitor of ferroptosis in various tissues and plays a crucial role in cell survival. Early studies demonstrated that *GPX4* deficiency induces ferroptosis in murine embryonic fibroblasts (49). Furthermore, *GPX4* interacts synergistically with the NF-κB signaling pathway to co-regulate apoptosis and ferroptosis in KGN cells (8). MNQ can modulate cell proliferation, differentiation, and apoptosis in Raji cells, potentially mediating the suppression of inflammatory responses, angiogenesis, and tumor metastasis via the NF-κB signaling pathway (50). In the present study, we observed that *GPX4* knockdown significantly reduced viability in sheep follicular GCs, an effect that D19 markedly attenuated. Overexpression of *GPX4* inhibits the NF-κB signaling pathway, thereby downregulating the expression of pro-inflammatory cytokines such as *TNF-α* and *IL-6*, ultimately protecting the rat heart from inflammatory damage (51). 5,8-Dimethoxy-1,4-naphthoquinone (DMNQ) derivatives significantly attenuated the LPS-induced expression of NO, ROS, and inflammatory cytokines in BV-2 microglial cells by modulating the MAPK/NF-κB signaling pathway (52). In agreement with these findings, our experiments demonstrated that D19 significantly mitigated the activation of the NF-κB pathway and the subsequent upregulation of pro-inflammatory cytokines (*TNF-α*, *IL-1β*, and *IL-6*) induced by *GPX4* interference in sheep follicular GCs. These results indicate that D19 treatment reversed this *GPX4* deficiency-mediated inflammatory response.

Oxidative stress resulting from *GPX4* deficiency significantly compromises reproductive efficiency in animals. *GPX4* is highly expressed in the testes and sperm, where it regulates spermatogenesis, maintains chromatin integrity, and counteracts oxidative stress to ensure male fertility (53). Its deficiency leads to abnormal sperm development, germ cell apoptosis, and reduced fertilization capacity (54), thereby significantly diminishing reproductive efficiency. Numerous studies have established *GPX4* as a transcriptional target of *NRF2*, with the two interacting to regulate cellular oxidative stress, ferroptosis, and iron metabolism (55). In HepG2 cells, LPS may induce oxidative stress by suppressing the activity of the NRF2/GPX4 axis, while in the D-GalN/LPS-induced acute liver injury model, MaR1 alleviates oxidative stress and ferroptosis-related liver damage by activating the NRF2/HO-1/GPX4 pathway, inhibiting ROS and MDA production, and increasing reduced GSH levels (56). Additionally, MNQ has been shown to inhibit glucose and LDH production in triple-negative breast cancer cells, concurrently suppressing cellular glycolytic activity and the expression of related molecules (32). In agreement with these findings, the present study demonstrated that D19 effectively ameliorated oxidative damage in sheep follicular GCs resulting from *GPX4* deficiency. Specifically, compared to the si-GPX4 group, D19 significantly upregulates antioxidant-related indicators (total antioxidant capacity, SOD activity, GSH-PX, CAT, and reduced GSH) in GCs, while downregulating ROS, MDA, glucose, and LDH levels. Consistently, *GPX4* exerts significant antioxidant activity within mitochondria. The upregulation of *NRF2*, *CAT*, and *SOD2* in mouse follicular GCs protects against premature ovarian failure (POF) (57). Our study revealed that D19 significantly enhanced the expression of *NRF2*, *GPX4*, *CAT*, *GSH-PX*, and *SOD2* at both transcriptional and translational levels in the si-GPX4 group. These findings highlight the crucial role of D19 in improving GCs oxidative damage through the NRF2/GPX4 pathway.

Mechanistically, iron overload impairs NRF2 binding to antioxidant response elements (AREs), resulting in downregulated *GPX4* expression, elevated mitochondrial ROS production, and diminished mitochondrial membrane potential, ultimately promoting lipid peroxidation and ferroptosis (58). Consistent with these prior observations, we found that the downregulation of *GPX*4 in sheep follicular GCs resulted in significantly elevated levels of lipid peroxides and Fe^2+^ concurrent with a marked reduction in mitochondrial membrane potential and the presence of abnormal mitochondrial morphology, all characteristic features of ferroptosis (59, 60). Importantly, the upregulation of *GPX4* has been shown to inhibit ferroptosis and delay the senescence of spermatogenic cells in aged mice (61). *FTH1* primarily functions to store intracellular iron and reduce free iron levels. As evidence, *FTH1* downregulation induces ferroptosis in bladder cancer cells (62). In the present study, si-GPX4 significantly decreased *FTH1* mRNA and protein expression levels in sheep follicular GCs, suggesting that *GPX4* depletion is sufficient to initiate ferroptosis in these cells. Research has shown that menaquinone-4, a form of vitamin K, exerts a protective role in a *GPX4*-deficiency-induced murine hepatocyte ferroptosis model by diminishing lipid peroxidation, cell death, and inflammation (63). Remarkably, we found that D19 effectively reversed ferroptosis in GCs caused by aberrant *GPX4* expression, highlighting a potential new approach for promoting GCs function.

Given our previous findings that D19 alleviates si-GPX4-induced ferroptosis and the associated inflammatory and oxidative damage in sheep follicular GCs, we hypothesized that D19 might also regulate si-GPX4-induced steroid hormone synthesis disorders. Evidence has established a close link between *GPX4*-mediated ferroptosis and steroidogenesis (64). *GPX4*, acting as a key antioxidant in testicular Leydig cells, safeguards steroidogenesis by modulating *STAR*-mediated cholesterol delivery to *CYP11A1* and the subsequent *CYP11A1*-catalyzed conversion of cholesterol into pregnenolone, ensuring normal testosterone secretion (65). Similarly, in our experiments, we observed that si-GPX4 significantly suppressed the expression of *CYP11A1* and *STAR*, along with other steroidogenesis-related genes (*HSD17B4*, *CYP19A1*, and *3β-HSD*) in sheep follicular granulosa cells, resulting in decreased E_2_ and P_4_ production. However, D19 treatment effectively reversed this si-GPX4-mediated steroidogenic dysfunction (17), potentially contributing to the maintenance of normal GCs function.

## 5 Conclusion

In conclusion, we synthesized the MNQ derivative D19 and systematically investigated its biological activities. Our findings indicate that D19 exhibits lower cytotoxicity than MNQ and effectively attenuates LPS-induced inflammation, oxidative stress, and steroidogenesis impairment, potentially via a mechanism involving the GPX4-ferroptosis axis. These results provide a theoretical foundation and a potential drug target for developing novel anti-inflammatory and antioxidant natural plant extracts to treat follicular developmental disorders and reproductive system diseases.

## Data Availability

The original contributions presented in the study are included in the article/supplementary material, further inquiries can be directed to the corresponding author.

## Glossary

MNQ: 2-methoxy-1,4-naphthoquinone
GCs: Granulosa cells
LPS: Lipopolysaccharide
qRT-PCR: Quantitative reverse transcription polymerase chain reaction
GPX4: Glutathione peroxidase 4
E_2_: Estradiol
P_4_: Progesterone
A_4_: Androstenedione
NO: Nitric oxide
OECs: Olfactory ensheathing cells
KGN cells: Human granulosa-like tumor cell line
ROS: Reactive oxygen species
POF: Premature ovarian failure
DMSO: Dimethyl sulfoxide
TNF-α: Tumor necrosis factor alpha
NF-kB: Nuclear factor kappa-light-chain-enhancer of activated B cells
IL-1β: Interleukin-1 beta
IL-6: Interleukin-6
CAT: Catalase
GSH-PX: Glutathione peroxidase
SOD2: Superoxide dismutase 2
NRF2: Nuclear factor erythroid 2–related factor 2
FTH1: Ferritin heavy chain 1
LDH: Lactate dehydrogenase
TEM: Transmission electron microscopy
MDA: Malondialdehyde
HSD17B4: Hydroxysteroid 17-beta dehydrogenase 4
CYP19A1: Cytochrome p450 family 19 subfamily A member 1
3β-HSD: 3 beta-hydroxysteroid dehydrogenase
CYP11A1: Cytochrome p450 family 11 subfamily A member 1
STAR: Steroidogenic acute regulatory protein
SA-β-Gal: SA-β-galactosidase
